# Species Richness Net Primary Productivity and the Water Balance Problem

**DOI:** 10.3390/e26080641

**Published:** 2024-07-28

**Authors:** Allen G. Hunt, Muhammad Sahimi, Erica A. Newman

**Affiliations:** 1 Department of Physics, Wright State University, Dayton, OH 45435, USA; 2 Mork Family Department of Chemical Engineering and Materials Science, University of Southern California, Los Angeles, CA 90089, USA; moe@usc.edu; 3 Department of Integrative Biology, University of Texas at Austin, Austin, TX 78712, USA; erica.newman@austin.utexas.edu

**Keywords:** ecology, percolation theory, net primary productivity, species energy theory

## Abstract

Species energy theory suggests that, because of limitations on reproduction efficiency, a minimum density of plant individuals per viable species exists and that this minimum correlates the total number of plant individuals *N* with the number of species *S*. The simplest assumption is that the mean energy input per individual plant is independent of the number of individuals, making *N*, and thus *S* as well, proportional to the total energy input into the system. The primary energy input to a plant-dominated ecosystem is estimated as its Net Primary Productivity (*NPP*). Thus, species energy theory draws a direct correspondence from *NPP* to *S*. Although investigations have verified a strong connection between *S* and *NPP*, strong influences of other factors, such as topography, ecological processes such as competition, and historical contingencies, are also at play. The lack of a simple model of *NPP* expressed in terms of the principal climate variables, precipitation *P,* and potential evapotranspiration, *PET*, introduces unnecessary uncertainty to the understanding of species richness across scales. Recent research combines percolation theory with the principle of ecological optimality to derive an expression for *NPP*(*P*, *PET*). Consistent with assuming S is proportional to *NPP*, we show here that the new expression for *NPP*(*P*, *PET*) predicts the number of plant species *S* in an ecosystem as a function of *P* and *PET*. As already demonstrated elsewhere, the results are consistent with some additional variation due to non-climatic inputs. We suggest that it may be easier to infer specific deviations from species energy predictions with increased accuracy and generality of the prediction of *NPP*(*P*, *PET*).

## 1. Introduction

A long-standing problem in ecology is the quantification of the relative importance of various factors that influence species number, *S*, including energy input [1], competition [2,3], history [4,5,6], and heterogeneity [7]. This well-known problem has been discussed by scientists as early as von Humboldt, Darwin, and Wallace [8,9,10]. The relative importance of these factors may not be universal but instead may depend on latitude [11,12], spatial scale [7,13,14,15,16], or the particular kind of organism under consideration [13]. Here, the discussion will be confined to the terrestrial plant species, and in particular, trees, for which evidence points to the dominant role of species energy theory, at least for sufficiently large spatial scales [13,17]. The fundamental hypothesis of species energy theory is that the number of species is proportional to the energy input into an ecosystem that, particularly for ectotherms, such as reptiles, may be expressed directly in terms of solar energy [13]. However, for plants, it appears that the dominant input energy pathway that is relevant to species number is the process of photosynthesis [17]. Total photosynthesis is estimated by Net Primary Productivity, *NPP*, which is limited roughly equally by water and solar energy [17,18]. As it has also been pointed out that the effects of these limitations on *NPP* are most parsimoniously expressed through the variable evapotranspiration, *ET* [18], it has thus also been suggested (and verified) that among climatic variables, plant species richness is primarily explained by *ET* [13] or by *P* + *PET* − *PET*^2^ (in both cases explaining 78% of variation) [13,17], where *PET* is potential evapotranspiration.

The purpose of this study is to determine whether or not new predictions of *NPP* based on percolation theory [19] can help to reduce what uncertainty remains in the role of species energy theory in species number *S* when *NPP* is represented as a function of climate variables. The basis of predicting plant ecosystem *NPP* from percolation theory is the success of predicting *NPP* (*R*^2^ = 0.97) with one universal adjustable parameter across all climate regimes [19].

The theoretical expression for *NPP* is developed based on the scaling relations of percolation theory for vegetation growth and soil formation in terms of the fundamental pathways for water, that is, evapotranspiration and run-off, combined with an optimality hypothesis that plant ecosystems with the greatest *NPP*, subject to all relevant constraints (climatic, edaphic, and so on), will tend to dominate [19,20]. Based on its key inputs, we refer to this optimization as the percolation optimality hypothesis. Although expressed in terms of ecosystem productivity, there is an advantage conferred upon individuals from their ability to intercept solar energy and reproduce themselves, which then may explain community dominance patterns. Maximizing ecosystem productivity by increasing niche and functional diversity, insofar as this increase is also consistent with maximizing the number of species [21,22,23], has received recent support [24,25]. This support addresses the concern in [13] that maximizing *NPP* could be accomplished by one or two dominant species.

The percolation optimality hypothesis can predict several quantities of potential interest, including (1) the energy input into an ecosystem as a function of climate variables; (2) the number of plant species present in such an ecosystem; (3) by inference, the prediction that the number of species present is proportional to the number of individuals (i.e., species energy theory) [1,13], the More Individuals Hypothesis [26,27]; and (4) by inference, the prediction that the average metabolic rate per individual is constant across climate regimes, even when the average energy per species is not. It is therefore also broadly consistent with earlier work showing that maximizing plant productivity in an ecosystem is possible when plant diversity is maximized [24,25,26,27,28,29,30,31,32]. Even if species diversity does contribute to ecological niche diversity [21], this diversity need not universally translate to genetic diversity, however. Although further discussion of the linkages of such distinct measures of diversity is undoubtedly required in the field of ecology, these areas of ongoing research are not further addressed here. 

Another potential application of the results presented in the present paper is the Maximum Entropy Theory of Ecology (METE). METE [28,29,30,31,32] exploits a minimum information concept (the maximization of information entropy) to derive distributions of species, individuals, and energy transformations in ecosystems. Although previous statistical models of ecological metrics have used Boltzmann entropy as a starting place, developments from Shipley et al. (2006) [33] moved the conversation in macroecology from a state of trying to more accurately measure and account for the variation of all climate and mechanistic variables across scales [14,15,16] to insights into how to use information entropy to better model complex ecosystems. METE similarly uses the more general Shannon entropy (−$\sum P_{i}{lnP}_{i}$) to predict the distribution of microstates from macrostates in an ecosystem in a dynamic steady state. The details of the treatment are beyond the scope of the present paper, but important inputs to the METE framework include the total numbers of individuals and species, as well as the total metabolic energy expended by an ecosystem, which the current approach can deliver, provided a steady-state has been reached. An extension of METE proposes an ecological equation of state, which unifies the relationships of species richness, productivity, abundance, and biomass in ecosystems. This equation shows a positive relationship between productivity and species richness, as mediated by the biomass and the number of individuals in the area under consideration. 

In the context of species richness, Currie [13] details the central role of evapotranspiration *ET* on *NPP*, and we therefore consider the relationship between *NPP* and *ET* in more depth. Rosenzweig [18] determined empirically that *NPP* was proportional to a power *s* of *ET*, whose value (1.69) was extracted from a global study across biomes. However, Hunt et al. (2024 and 2021) [19,34] demonstrated that the actual value should be related to the root mass fractal dimension. Assuming the roots follow fractal paths of least cumulative resistance, *NPP* should more accurately equal the relevant mass fractal dimension predicted by percolation theory, which quantifies the topology of such paths [35]. Since, according to [36,37,38], dominant rooting depths appear to be relatively shallow (less than half a meter, a meter, or 2 m, respectively), it was assumed that the relevant percolation mass fractal dimension should be its value in two dimensions (2D) [35], i.e., 91/48 = 1.896 (obtained through exact calculations and exact scaling relationships). In order to predict the proper dependence of *NPP* (*ET*) on climate variables, *P* and *PET*, however, it becomes necessary to determine *ET*(*P*, *PET*), which is a problem known in hydrology as the water balance and viewed as a central problem of that field of study [39,40,41].

The water balance quantifies how much precipitation goes to evapotranspiration *ET* and how much runs off, equal to *P* − *ET* [39,40,41]. Measurement of the water balance is often more easily obtained through measurement of the streamflow exiting a drainage basin than through direct measurement of *ET*; however, *ET* is not related conceptually to the specific scale of a drainage basin except in cases of significant relief [17]. We show how the water balance is constructed out of soil formation (see, e.g., [42]) and vegetation growth (e.g., [43]) rates and give an overview of how the proposed ecological optimality can be used to find the partitioning of surface water on the terrestrial Earth surface that leads to maximum productivity. The process is based on a scaling relationship for solute transport that describes soil depth evolution [42] and one for optimal paths that describes the distinction between root growth rates and root lateral extent [43]. The results have been shown to predict plant productivity as a function of climate variables [19] and are shown here to provide accurate predictions of species richness in terms of the same climate variables. It is also noteworthy that the result obtained for *ET* (*P*, *PET*) from maximizing ecosystem *NPP* is almost identical to one obtained from a dynamic process model that maximizes carbon profit [44].

Before continuing, two points should be mentioned. First, the formation of biomass from sugar involves a set of complex processes using sugar for energy (metabolism), which are not considered further here but help differentiate between carbon assimilation [19] and carbon profit [44]. Second, it is important to remember that the word evapotranspiration is constructed, in principle, from a sum of the effects of two rather different processes: evaporation of water from bare ground or plant surfaces and the process of transpiration by which water evaporated from the stomata of leaves, thereby drawing further water from the ground into and through the plant.

## 2. Theoretical Background

*NPP* is limited by multiple factors. In view of its dependence on the process of photosynthesis, sugar is constructed from water and carbon dioxide through the absorption of photons. Therefore, solar energy, atmospheric carbon, and water may all be limiting components, as well as soil nutrients. However, atmospheric carbon contents, though they change significantly over time, are essentially spatially uniform in comparison with the climatic inputs [17,18]. Spatial variability in the carbon cycle is critical at any particular time and is, thus, due in large part to the spatial variability of climate variables, precipitation *P,* and potential evapotranspiration, *PET* (a measure of solar energy). Because water drawn from the soil requires water evaporation from stomata, the indirect demand for plant-transpired water is higher than the direct demand from photosynthesis. However, an insufficient supply of either water or solar energy will suppress photosynthesis and, thus, plant productivity. It was proposed nearly 60 years ago [18] that both limiting components of the carbon cycle could be accounted for using a single variable, evapotranspiration, *ET*. More recently, it has been proposed [19,34] that the soil depth should be a second input into the dependence of *NPP* on *ET*. The soil depth also relates to percolation theory.

Roots grow primarily within a thin soil layer, variously estimated as 0.5 m, 1 m, or 2 m [36,37,38]. Compared with typical drainage basins, or catchments, which are measured in kilometers or greater, this suggests that the root zone may, over a wide range of scales and conditions, be considered nearly two dimensional (2D). The 3D root zone has also been considered and is doubtless relevant at some scales [19]. In addition, for a variety of reasons, roots have been proposed to follow optimal paths [43]. In a model that treats such paths in the spirit of percolation theory, root masses occupying such paths have been shown [43] to form self-similar fractal structures with a fractal dimension *d_f_* that is nearly equal to that of the largest 2D percolation cluster [35], *d_f_* = 91/48 = 1.9.

It is argued [35] that flow in disordered porous media is dominated by the contribution from connected paths near the percolation threshold and, thus, universal power law scaling of percolation theory is relevant. The time required for solute introduced at a single source (such as from a reacting soil particle) at one side of a system to reach the other side was shown [45] to be proportional to the length of the system to a power equal to the fractal dimension of the percolation backbone, *D*_b_, the multiply connected part of the percolation cluster in which fluid flow and solute transport occur (as the rest of the pores are dead ends). For conditions appropriate to weathering, i.e., either wetting soils or constant moisture content, *D*_b_ was determined [46] to be equal to 1.87. Validity of the advection-dispersion equation at the scale of an individual pore implies equality of water and solute velocities at that scale and requires a spatio-temporal scaling relationship for the solute’s distance traveled, given by x_s_ = x_0_ (*t*/*t*_0_)^1/D^_b_, where *x*_0_ is a pore separation, or typical particle size, and *x*_0_/*t*_0_ = *v*_0_ is, in natural ecosystems, a yearly mean pore-scale flow rate [43]. The result is

(1) $$x=x_{0}{\left(\frac{t}{t_{0}}\right)}^{1/1.87}$$

Because solute transport tends to be the primary limiting factor in chemical weathering, which itself is the principal limitation to soil formation [47], Equation (1) gives the soil depth, while its time derivative, *dx*/*dt*, predicts the soil production function. The steady-state (constant) soil depth is defined by the equality of soil production and the rate of soil removal *D*_0_. Equating *dx*/*dt* with *D*_0_ yields [42]

(2) $$x=x_{0}{\left[\frac{P-ET}{1.87\phi D_{0}}\right]}^{\frac{1}{D_{b}-1}}$$

where porosity ϕ is necessary to convert a mean atmospheric velocity—or flux—of precipitation *P* less evapotranspiration *ET* to a water velocity in the porous medium. Since only water flowing through the soil into the subsurface can contribute to weathering and soil formation, the flux of water returned to the atmosphere through evapotranspiration *ET* does not contribute.

A broadly analogous argument provides a relationship for vegetation root growth [43] with the same form as Equation (1), but with one significant difference: the exponent in the power law is predicted from percolation theory to be 1/*D*_opt_, which, by considering the root zone to have shallow depth, is taken to be the value [46] appropriate for 2D, i.e., *D*_opt_ = 1.21. The explanation is shortened here. By applying a gradient in the soil water potential, the roots draw in water, which can intersect nutrient sources. Water flow toward the roots is dominated by the optimal paths of percolation. Accordingly, in a search for nutrients and water, root tip extension tends to follow optimal paths ’back up-gradient toward nutrient sources. Thus, the actual root length *l* is longer than the root radial extent, x_r_, due to the tortuosity of the paths with the smallest cumulative resistance, and *l* = (*x*_r_/x_0_)*^Dopt^*. Since root tip extension rates tend to be a property of genetics plus nutrient and water availability [48], *l* has no obvious scale dependence, making *l* = v_0_ *t*, and

(3) $$x_{r}=x_{0}{\left(\frac{t}{t_{0}}\right)}^{1/1.21}$$

Although the length scale and flow rates for both soil formation and root growth are represented identically, the meaning of x_0_ in Equation (3) is, instead of being a particle size, a typical plant xylem diameter, while v_0_ is a little larger for plants, since they will be seen to take almost twice as much of the water available is left for flowing through the soil. Nevertheless, these particular distinctions have no effect on the vegetation optimality condition or the predictions of the percolation model described here. On an annual scale, the water evaporated off plant stomata is represented as the transpiration.

The production of biomass relates to the mass of an object, such as the roots. The root architecture can be modeled as a fractal (e.g., [37]). For consistency in approach, the choice of a 2D percolation exponent for the optimal paths’ tortuosity requires the use of the 2D percolation exponent for the root mass fractal dimension. Using the argument that the root mass will depend on its lateral extent, it was then proposed [43] and references therein that *NPP* should be proportional to, *x*_r_^1.9^, i.e., the lateral spread raised to the power of d_f_ = 1.9. The result is the expression *NPP* = *c ET*^1.9^, with *c* being a constant. Here, transpiration, which is typically [49] approximately 2/3 of evapotranspiration, *ET*, was approximated as *ET*. However, the total mass in the root system must also be expressed in terms of the vertical dimension. Thus, the yearly increase in biomass is proportional to the product of root depth and horizontal contribution to the biomass (*ET*^1.9^). 

Since *NPP* has an *ET*^1.9^ dependence and soil depth x_s_ depends on *P* and *ET* as (*P*-*ET*)^1.15^, it is straightforward to determine the maximum of *NPP* by differentiating the product *ET*^1.9^ (*P* − *ET*)^1.15^ with respect to *ET* and setting the resulting equation to zero. Excluding the trivial endpoint solutions, *ET* = 0 and *ET* = *P* (allowing division by *ET* and *ET* − *P*), the resulting equation is 1.9 *ET* = (1.9 + 1.15) *P*. The result [19] is a proportionality of *ET* to *P* with a proportionality constant involving only universal exponents from percolation theory [35] and a value 0.623; thus, *ET* = 0.623 *P*. The second derivative of *NPP* with respect to *ET* is negative at *ET* = 0.623 *P*, demonstrating that this particular value of *ET* generates a maximum; however, it is much easier to determine that the extremum is a maximum by applying physical arguments. Since annual *NPP* cannot be negative if plants even exist, *ET* can neither exceed *P* nor be negative. This argument is also consistent with a physical argument that *ET* can only take on values somewhere between 0 and *P*. But, at the endpoints of the domain, when *ET* = *P*, or when *ET* = 0, the above expression for *NPP* yields 0. Therefore, a maximum must exist for some *ET* value between these two limits.

The above optimization procedure must be extended when there is a deficit of either water or energy, i.e., *P* ≠ *PET*, with *PET* defined explicitly as the depth of water that would evaporate off a free surface in one year. An index called the aridity index, *AI* = *PET*/*P*, has been proposed to distinguish between the water and energy limitations. For water-limited systems corresponding to *AI* > 1, the same optimization procedure was applied only to that fraction of the ground covered by vegetation (which is proportional to *AI*^−^^1^), with *ET* elsewhere approximated as being equal to *P*, and for energy-limited systems, with *AI* < 1, the optimization procedure was applied only to that fraction of *P* that could be evaporated (i.e., equal to *PET*) [19], and the remaining *P* contributing to run-off.

The results of the optimization, including energy and water limitations for *ET* (*PET*,*P*), were then substituted back into *NPP* = *c ET*^1.9^ to obtain two equations for aridity indices higher and lower than 1 [19].

(4) $$NPP=c\;{\left(0.623\;PET\right)}^{1.9\;}\;AI\leq 1$$

(5) $$NPP=c{\left[P-0.377\frac{P^{2}}{PET}\right]}^{1.9}\;AI>1$$

For *P* = *PET* (*AI* = 1) the two expressions are identical and equal to c (0.623 *P*)^1.9^. In Equations (4) and (5), a single unknown parameter, *c*, is used to convert units of precipitation in mm^1.9^ yr^−1.9^ to units of productivity, gC m^−^^2^ yr ^−^^1^. Assuming the validity of species energy theory (supported by Refs. [13,17], with further evidence of a direct proportionality of *S* and *NPP* [28,50]), Equations (4) and (5) can now be utilized to predict species richness as well. In such a procedure, an additional multiplicative parameter of unspecified magnitude with units of m^2^ yr gC^−^^1^ must be utilized in order to obtain results that are unitless. The two parameters may be combined into a single parameter, *h*, with units of yr^1.9^ mm^−^^1.9^. Effectively, this gives the product of the number of species per gram of carbon biomass produced (per year per meter), times the biomass produced per unit of evapotranspiration. Doing so does, however, somewhat obscure the (assumed) central role of *NPP* in the determination of species number *S*. 

It was shown in percolation optimality theory (Hunt et al. 2024) [19] that, by comparison with a data summary of Budyko (1974) [40], the constant *c* may be treated as universal across climate regimes with *PET* varying by a factor of 7, and for each separate *PET* value, the aridity index AI varying by 2 orders of magnitude. The direct comparison [19] of all the values in [40] with Equation (4) resulted in an *R*^2^ value of 0.97, assuming a universally relevant value of *c*. However, the combined factor relating climate variables to *S* is different, depending on what subset of the total number of plant species is considered, with the largest value of the constant appropriate for all plant species, a smaller value for the number of vascular plants, and a yet smaller value if only tree species are meant. Because of their high production of biomass, expressions for tree species number *S* tend to require a larger *NPP* per species, with fewer individuals in a given area and a correspondingly smaller number of species. Other variation in *h* is expected to be traceable to other inputs to species richness, such as heterogeneity (e.g., topography) for a given scale, geologic or climatic history, or variation in scale.

## 3. Principal Data Sources

We chiefly use data from Currie, 1991 [13], who reported the dependence of tree species richness on climatic data across North America north of Mexico. He divided up this region into lat-lon quadrats of 2.5° by 2.5° south of 50° N and of the same width in longitude, but double the height in latitude, north of 50° N. Thus, not all regions were of the same size, which introduces an uncontrolled variation in *S* of up to a factor 2 that is not generated by climate, with the justification of increased accuracy in other regards. To count the number of species in each quadrat, maps of the ranges of tree species were overlaid with one another. For each quadrat, Currie determined annual mean values of *P* and *PET*, as well as *ET* (and other climate variables), referring to the methods of Budyko [40]. At 70° N, roughly tundra latitude, *PET* dropped to under 100 mm yr^−^^1^ at ca. 50 mm yr^−^^1^, marking the highest latitude and smallest *PET* values where trees were found. Quadrats completely covered with tundra had no tree species. The largest value of *PET* was about 1800 mm yr^−^^1^, while that of *P* was almost completely restricted to 1600 mm yr^−^^1^. Currie [13] also calculated *NPP* but did not represent *S* in terms of *NPP*. Latham and Rickfels (1993) [5] augmented the data set of tree species numbers from [13] by including additional compatible data for South America, Eastern Asia, and Japan. The purpose of their investigation was to determine whether the greater continuity in climate in Eastern Asia through the glacial episodes of the Pleistocene could contribute significantly to a greater tree species richness, *S*. The data for South America were expressed as ranges of values, not strictly compatible with the data of [13], and we did not incorporate them.

## 4. Comparison with Data

**Figure 1:**
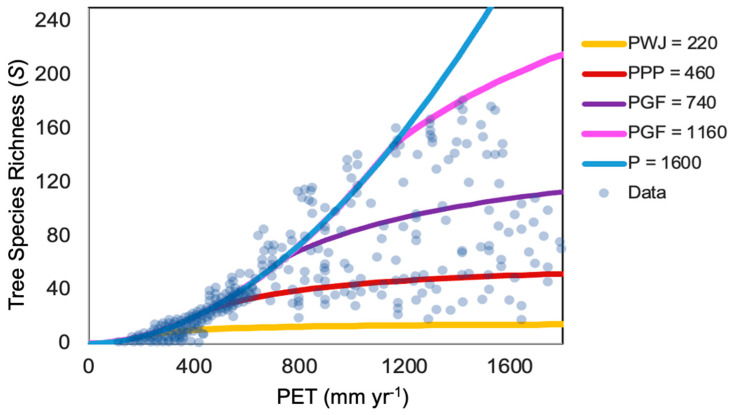
Comparison of predicted tree species number, *S*(*PET*), for various values of *P* with observed numbers of tree species as a function of *PET*. The largest value of *PET* reported was 1800 mm yr^−1^. PWJ 220 (mm yr^−1^) refers to the lowest rainfall boundary where Western Junipers are reported [52] in Eastern Oregon, which approaches the lowest elevations along the valley floors [53]. PPP = 460 refers to the lowest *P*, where Ponderosa Pines are reported [52]. PGF = 740 refers to the smallest value of P for which Grand Fir is reported. PGF = 1160 refers to the largest value of *P* for which Grand Fir is reported. *P* = 1600 refers to the upper precipitation limit on significant data (a few additional points are found in the vicinity of 2000 mm yr^−1^). Because the Canadian North data are severely restricted in *PET*, the range 0 < *PET* < 400 mm yr^−1^ is treated separately below, while the guidance given for Eastern Oregon is more appropriately restricted to *PET* > 400 mm yr^−1^.

**Figure 2:**
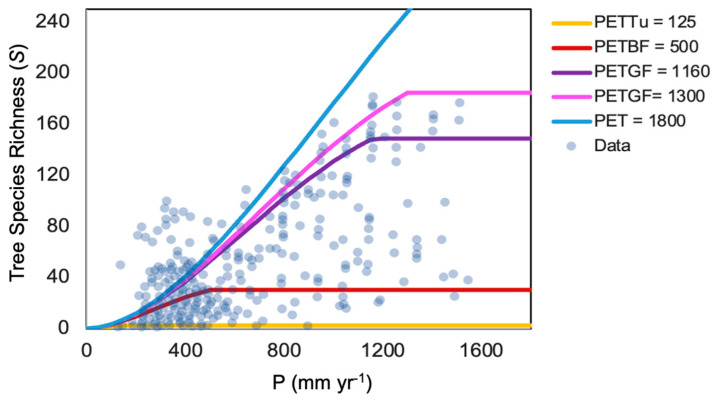
Comparison of predicted tree species number, S, as a function of P for various values of *PET*. PETTu = 125 corresponds to the Arctic Circle, a proxy for the onset of the tundra. *PET* 1800 corresponds to the largest *PET* values found in Figure 1. The other values are labeled analogously to Figure 1, with GF referring to the smallest and largest *PET* values bounding the habitat of the Grand Fir and the label BF meaning Boreal Forest.

Currie [13] carefully investigated climate severity, climate variability, glacial history, heterogeneity, and energy as potential influences on species richness and concluded that energy was the strongest predictor, with variations in *ET* accounting for 78% of the variability in *S*. He went on to discuss that, although correlations do not demonstrate causality, they do serve useful functions, i.e., “when a correlation predicted by a hypothesis is weaker than other observed correlations, one may conclude that a better hypothesis exists”. Using this guidance, we tested Equations (4) and (5) with a constant *c* independent of climate variables to investigate the accuracy of the percolation optimality model of *NPP* for predicting the species number (Figure 1). Because Currie (1991) [13] does not give both *P* and *PET* values for the same site, it is not possible to compare predictions for each site directly without the original data and generate a statistical evaluation. In their comparison with data from [40], Hunt et al. [19] apply Equations (4) and (5) in terms of a family of curves *NPP* (*PET*/*P*, *PET*), each as a function of *AI* = *PET*/*P* for different values of *PET*. The separate representations of *S*(*PET*) and *S*(*P*) in [13] require developing an alternate procedure with a coordinated comparison between the two. Thus, the value of the constant *c* is the same in both comparisons, while the range of *PET* values shown in *S*(*PET*) must be used as the source of distinct *PET* values chosen for comparing with *S*(*P*) and vice versa. Thus, like Currie, we investigate correlations between *S* with *PET* (Figure 1) and *P* (Figure 2) separately for the entire data set. 

**Figure 3:**
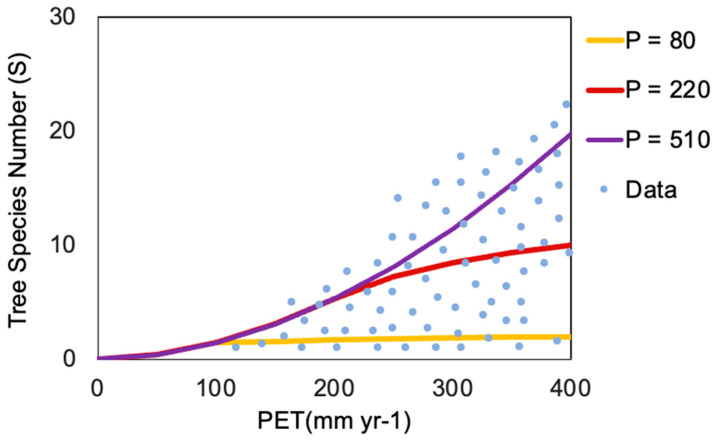
Comparison of predicted tree species number, *S*, as a function of *PET* for ranges of *P* and *PET* appropriate for the Canadian Northwest of Hudson Bay. The area between the yellow (*P* = 80) and the purple (*P* = 510) curves is where data are expected. The intermediate value (brown curve, *P* = 220) forms a lower bound for measured tree species at much larger values of *PET* in, e.g., Eastern Oregon and represents the driest climate there for Western Juniper but has no special relevance in the Canadian North, where *PET* does not exceed 400 mm yr^−1^.

In addition to the paired test, it is possible to augment the visual test of the predictions for *S*(*PET*) by separating different geographic scales. This is accomplished by focusing on the data for the major provinces of the Canadian North (Nunavut, Yukon, and Northwest Territories) since this region, with the southern boundary at 60 degrees N, accounts for the *S* values in Figure 1 with *PET* < 400 mm yr^−1^ (Figure 8 in Ref. [13]). In Statistique Canada (2022) [51], Average Annual Precipitation, by Ecoprovince (1979–2006), all P values in these three provinces fall within the range of 80 mm yr^−1^ to 510 mm yr^−1^. Applying these values of *P* for *PET* < 400 mm yr^−1^ leads to the results shown in Figure 3.

The author [13] investigated various other organisms (birds, reptiles, and so on) as well but found a monotonic relationship with climate variables only for tree species. The author noted a strong correlation with any of the energy variables, but the strongest was with *ET*. The relationship with *P* was described as “weak”. It is important to note, however, that the slope of the upper bound on species richness for *S*(*PET*) is not a monotonic function of *PET* and that our prediction generates the observed change in curvature. 

In Figure 1, Figure 2 and Figure 3, the value of the constant *h*, which converts units of *ET*^1.9^ to the unitless *S*, is maintained at 0.0055 yr^1.9^ mm^−1.9^.

In Figure 1, the smallest *PET* with a non-zero *S* is *PET* = 117 mm yr^−1^ with *S* =1. For smaller *PET*, *S* appears to be 0. In Figure 2, the smallest *P* reported is 128 mm yr^−1^, likewise with *S* = 1, and converging to 0 for smaller *P* (though one point at 138 mm yr^−1^ has *S* = 49). Equations (4) and (5) yields *S* = 1.4 for *PET* = 100 mm yr^−1^ and *P* = *PET*, and equally for *P* = 100 mm yr^−1^ and *PET* ≥ *P*. For Figure 2, we chose *PET* = 125 mm yr^−1^ to denote the onset of the tundra for the reasons described next.

*PET* = 50 mm yr^−1^, for which Equation (4) predicts *S* = 0.4 < 1, occurs at (70 °N) (Figure 8A in Ref. [13]), which skims the northern edge of Canada west of Hudson Bay (and marks the *S* = 0 contour in Figure 1A of [13]). At the Arctic Circle, values of *PET* range from about 50 mm yr^−1^ to 200 mm yr^−1^, with a mean of 125 mm yr^−1^, which generates *S* = 2, barely distinguishable from 0 in Figure 2. On the tree species map of Canada (Figure 1A of [13]), *S* drops below 10 near the Arctic Circle, but not to 2. However, for *P* and *PET* both 200 mm yr^−1^, within the range of *PET* values shown in Figure 8A of Ref. [13], the calculated value of *S* is 5.

It is important to note that a *PET* of approximately 500 mm yr^−1^ (and for equal precipitation, calculated *S* = 30) occurs just north of the latitude 50° N [54], which corresponds approximately to the onset of the boreal forest. Starting at approximately this latitude, the contours of species follow latitudinal boundaries rather closely across Canada, with *S* between 30 and 40 at 50° N. As can be seen, for such latitudes with strong energy limitations, *PET* describes most of the variability in *S*, both in the figure and on the map. This characteristic is shared in a global data set of vascular plants addressed below. 

In Figure 1, within the range 1160 < *PET* < 1300, which corresponds to the range of the Grand Fir [52] in *PET*, PGF = 740, at the dry end of the range of Grand Fir in *P*, approximately matches the largest *S* values in the Western USA that varies from 80 to 100. However, PGF = 1160, which generates the upper limit of the *P* range of Grand Fir, corresponds to *S* = 160 or larger, almost double the maximum number of species in the west, and a better match for the largest values of S found in the Southeastern USA at similar *PET*.

Consider Figure 2 again. Since the *PET* ranges for the Western Juniper and the Ponderosa Pine range from 1300 mm yr^−1^ to 1610 mm yr^−1^, but the *p* values vary from 220 mm yr^−1^ to 760 mm yr^−1^, their ranges are compressed on this figure to lie between the purple and the light blue curves, where these curves are adjacent. The Grand Fir, however, is constrained to lie between the purple and the magenta curves on the left side of the plateau in the purple curve. Again, this puts the Grand Fir in the region where between about 130 and 160 species are expected, but only 80–100 are found. 

Currie [13] explains the existence of the cloud of points outside the range of expected *S* values (above the 1600 curve) as tracing to the higher-than-expected species richness in the (arid to semi-arid) Southwestern USA. Strong heterogeneity in topography and water resources over relatively small spatial separations is likely the cause here, as was demonstrated through Currie’s correlation between heterogeneity and richness. The interpretation of the overall results is also enhanced by an examination of Figure 1 in Currie's work [13], which shows local minima in *S* < 30 in the rain shadow of the Sierra Nevada (Great Basin) and in two regions along the Southern High Plains with *S* < 20. Juniper habitats are found in both of these relatively arid regions. Sufficient heterogeneity exists to place juniper habitat into the same quadrat with nearby habitats that support more (and less) trees, but not on as fine a scale as in California, where tree species numbers are everywhere above 40 and mostly above 60. Thus, topographic effects help to limit the number of tree species (Figure 1) at the lower range of WJ to 20 rather than 2 (which would reflect the local association with pinon pine). Thus, the map helps in understanding the cut-off in data at P of approximately 220 mm yr^−1^ for a wide range of *PET* with a fairly large *S* value (near 20).

Although we do not show the data from Kreft and Jetz (2007) [55] for the global vascular plant species number as a function of *PET*, the authors report that their data reflect a strong correlation of species number *S* with *PET* for *PET* < 500, but little predictability solely from that climate variable at larger *PET*, similar to the result found by Currie (1991) [13], although, in that case, the strong correlation extends to *PET* > 600. These observations likely have the same cause, namely, that for sufficiently large *PET*, omission of consideration of the variability in *P* misses most of the predictability from climate variables.

**Figure 4:**
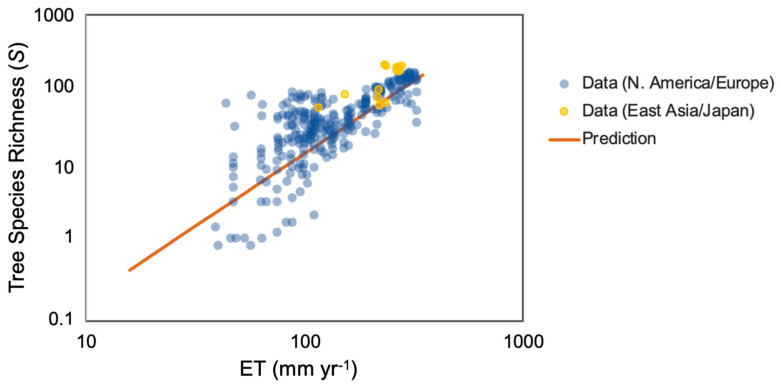
*NPP,* predicted as proportional to *ET*^1.9^, is compared with species richness data from North America and Europe [13] and separately from Eastern Asia and Japan [5].

Figure 4 demonstrates that similar climates (Eastern Asia [5] and North America-Europe [13]) with identical values of *ET* and, thus, to the lowest approximation of the same *NPP*, may have different numbers of tree species. The enhancement factor in Eastern Asia is approximately 2. The explanation for this discrepancy is given by Ref. [5] as related to natural history. Thus, North America and Europe, covered in the relatively recent geological past by ice sheets, may have fewer species since, in contrast to Eastern Asia, sufficient time has not elapsed for the ecosystem with the maximum number of species to develop. Currie [13] appears to have anticipated this argument, investigating North America for a correlation between *S* and Pleistocene glaciation, and found none. His interpretation was that a time span of over 10,000 years was adequate to remove the signs of past climate within the vegetation. However, Pleistocene climates affected strongly all of North America north of Mexico, so perhaps such an evaluation misses a critical input. If the interpretation of Ref. [5] is correct, the Shannon entropy associated with Asian ecosystems would be somewhat larger. Thus, maximizing Shannon entropy would, similar to conventional statistical mechanics, eliminate information that would help establish the history of a system. In the particular case discussed related to Holocene climate change, the simplest calculation, replacing each probability that a tree (in N. America) belongs to a given species by half its value (twice the value of *S* in E. Asia), would result in an increase in Shannon entropy of ln (2). In the event that this is a non-equilibrium value, that difference would then be expected to disappear with sufficient time. If, however, the difference is due to, e.g., a greater relief (heterogeneity), then the distinction would tend to remain.

In any case, the slope of log *S* vs. log *ET* is compatible with the percolation prediction, 1.9 in both cases.

## 5. Discussion

Our predicted results for *NPP*, known to accurately forecast [19] plant productivity when compared with the classic data set [40], also track species richness rather closely, including the precipitation and potential evapotranspiration dependencies individually. Using a universal constant (for North America, at least), the observed regular dependence of tree species number on latitude over the northern half of the continent was predicted. In the US, representative species (Grand Fir, Ponderosa Pine, and Western Juniper) showed up in approximately their appropriate positions in the *NPP* (*P*, *PET*) space (equivalent to *S* (*P*, *PET*) space with a single constant of proportionality). The resulting proportionality of *S* to *NPP* is consistent with the species energy theory, which makes *S*, *N*, and energy input all proportional to each other. 

The basis for the theoretical predictions lies in a new theory of the water balance [19] and the associated carbon cycle, itself developed using percolation scaling for growth, productivity, and soil formation, and the principle of ecological optimality. The fundamental basis for the optimality chosen here is similar to that proposed originally in Ref. [20] in its assumption that a relationship between *NPP* and water fluxes can be used as an objective function to optimize maximum productivity. However, in Ref. [20], the optimization was internal (i.e., within plants). Here, the optimality expresses a competition for the water between plants and soil together with the mostly symbiotic relationship between soil and plants. If the plants do not receive any water from precipitation, they cannot grow and enrich the soil with carbon and such nutrients as nitrogen, nor can the organic acids they otherwise produce initiate chemical weathering of the subsurface, and the water flux can at most transport carbonic acid from the atmosphere. Thus, a necessary precursor to soil development is completely missing in this particular scenario. On the other hand, if the plants take all the water from precipitation, none passes by to take the organic acids or nutrients down into the subsurface, and no soil is produced. Since most plants scarcely grow without soil, there must be a maximum in productivity somewhere in between, i.e., 0 < *ET* < *P* (a point summarized in a University of Texas Dallas Geonews video: https://www.youtube.com/watch?v=xv-n54NTd9M (accessed on 23 July 2024)).

Determining the optimum in the water balance (*ET*) through maximization of *NPP* allows the prediction of *NPP* as a function of climate variables. The maximization of *NPP* may require maximum diversity [22], which often implies the need for sufficient time under a stable climatic regime for all relevant ecosystem members to adapt or be transported into the region studied. However, disturbance regimes may strongly influence these patterns in the double role of potentially generating diversity over long time scales and taking up *NPP* (as through wildfire) [56]. Our approach is in general accord with the advice of Whittaker and Field (2000) [57]: “Predictable global patterns in species richness at the macro-scale can be accounted for by a simple climate model, based not on actual evapotranspiration (AET), which is an ambiguous variable, but on potential evapotranspiration (PET) and annual rainfall […] Historical contingency provides an important part of the explanation for residual variation, left over when climate has been accounted for.” We now check whether our results can help with the analysis of the role of historical contingency in the distinction between Asian and North American tree species richness.

Using [58], the maximum values of *PET* = 2000 mm yr^−1^ calculated by the Thornthwaite (1948) [59] or Penman–Monteith (1965) [60] method or *PET* = 2200 mm yr^−1^ calculated by the Priestley–Taylor (1972) [61] for tropical conditions (NDVI between 0.6 and 0.8), Equations (4) and (5) for *P* ≥ *PET* yields values of *S* equal to 418 and 501, respectively. Accounting for the ca. factor of 2 increase in *h* for Eastern Asia (Latham and Rickfels, 1993 [5]), as compared with North America, would generate maximum values of 836 and 1002 for *S*. Notably, these two values are close to the largest recorded values in the tropics (1000 < S < 1500) in both the Americas and Southeastern Asia (though at much smaller scales) in [62]. The greater continuity of the predictability of species richness in Eastern Asia as opposed to the significant disconnect in the Americas is consistent with the assertion of Latham and Rickfels (1993) [5] regarding the role of history (“tropical conservatism”), as well as the argument of Whittaker and Field [57] regarding the importance of an accurate representation of the primary, i.e., climate, input to species richness in determining the relative importance of secondary inputs. It is also possible that greater heterogeneity in topography in Eastern Asia, with its extreme relief, might have contributed to the greater diversity found in the Latham–Rickfels study [5]. 

## 6. Conclusions

We presented a proof of concept for applying the hydrological principles of water balance, the near-2D development of a soil layer across the landscape, the fractal-like development of root structure (following the logic of percolation theory), and the water requirements of plants to link potential evapotranspiration with species richness. We tested this prediction with previously studied data [13] and found realistic trends for the entire data set and for several individual species. In a later extension of the range of PET values investigated to values consistent with the maximum observed PET in the tropics, we discovered that a single formula (Equations (4) and (5)) generates values of S consistent with the observation from the Arctic to the Equator and helped to understand quantitatively the distinction between Asian and N. American tree species richness consistent with the tropical conservatism hypothesis. 

This work aims to fill a gap in existing knowledge, that is, how species richness is mechanistically related to climate variables and water balance through constraints on water use by transpiration by plants and spatial constraints on their root growth. It also highlights important questions in biodiversity sciences as to what constrains overall species diversity. It provides an energetic justification/mechanism for the formation of richness that was already mentioned in previous work (e.g., [63]). Future work may delve more deeply into additional data sets or link this percolation optimality framework with the Maximum Entropy Theory of Ecology through the prediction of species richness. With such climate-related and mechanistic constraints, it may be possible, through the use of macroecological theory, to develop a much more complete picture of constraints on species diversity and productivity at local and global scales. 

Important aspects missing from the present formulation include that it does not address the role of ecological networks [64], for which the data used in the present study have no temporal dependence. Without any means to address temporal dependences, our study therefore omits a priori potential causal inputs. Given the definition of geographic regions defined by the author [13] of the tree study in terms of a global coordinate system (latitude and longitude), it also seems to be impossible to expand this particular study to address the potential effects of drainage basin architecture and river corridor correlations in species numbers, even though the importance of such inputs is known [65,66]. In contrast to the impossibility of modifying the present study to address such important inputs into the temporal and spatial variability of species numbers, the results of our study may be relatively easily incorporated into (functional or geographical) network analyses of (plant and animal) species richness patterns and their development through time. For, as has long been known, animal species patterns should be linked to the plants with which they are associated [67].

## Data Availability

Data used in this paper are available in the original articles in which they appeared.
